# Reproductive Senescence in Drones of the Honey Bee (*Apis mellifera*)

**DOI:** 10.3390/insects10010011

**Published:** 2019-01-08

**Authors:** Bradley N. Metz, David R. Tarpy

**Affiliations:** Department of Entomology & Plant Pathology, NC State University, Raleigh, NC 27695, USA

**Keywords:** *Apis mellifera*, drone, sperm viability, senescence, aging, reproduction, spermatozoa, honey bee

## Abstract

In the face of high proportions of yearly colony losses, queen health and fecundity has been a major focus of industry and research. Much of the reproductive quality of the queen, though, is a function of the mating success and quality of the drones (males). Many environmental factors can negatively impact drone semen quality, but little is known about factors that impact the drones’ ability to successfully mate and deliver that semen, or how widely drones vary. In our study, we observed the daily variation in honey bee drone reproductive quality over time, along with a number of morphological traits. Drones were reared in cages in bank colonies, and 20 individuals were dissected and measured daily. The number of viable spermatozoa in the seminal vesicles was zero at emergence and reached an average maximum of 7.39 ± 0.19 million around 20 days of life. Decline in spermatozoa count occurred after day 30, though viability was constant throughout life, when controlling for count. Older drones had smaller wet weights, head widths, and wing lengths. We predict that this is likely due to sampling bias due to a differential lifespan among larger, more reproductively developed drones. Our study shows that drones are more highly variable than previously suggested and that they have a significant variation in reproductive physiology as a function of age.

## 1. Introduction

### 1.1. Pollinator Decline

The past half-century has seen a major decline in the numbers of managed honey bee colonies [1,2,3,4] and, more recently, there has been documentation of severe colony losses, first gaining wide notice as colony collapse disorder in 2007 [5]. Colony losses have continued in subsequent years, with US beekeepers reporting an average of 40.5% of their colonies lost every year [6,7]. These trends of increased mortality also occur more widely in other insect pollinator taxa [8,9,10]. Although the number of managed colonies of honey bees has largely remained stable over the past decade, the population is sustained by the continual need of beekeepers to replace colonies and a corresponding increase in their management costs [4]. This, combined with increased human reliance on pollinator-dependent crops [11], has the potential to lead to increased economic hardship across all levels of food production [12,13].

### 1.2. The Importance of Queens

In determining the underlying causes of the high rate of yearly attrition in honey bee colonies, much attention has been focused on queens [14,15]. As the sole reproductive female of the colony, the queen is the most visible individual when assessing colony reproductive output [16] and a primary focus of beekeepers. This has culminated in an industry-standard practice of replacing queens yearly—well below her maximum reproductive lifespan under ideal circumstances [17]. There is now a broad consensus that queen health and fecundity is extremely important for the health and productivity of the colony [17,18,19,20,21,22,23,24,25]. Queen reproductive quality is defined not just by her potential fecundity (largely a function of body size), but on her mating success as well.

Queens rely on multiple drones (honey bee males) to inseminate them with high-quality sperm on one or more mating flights [26,27], which they then store in their spermathecae and fertilize eggs as they lay them. A queen’s lifespan is dependent on her ability to lay fertilized eggs, and the colony will undergo the risky process of queen replacement to maintain its life beyond that of a single queen [16]. Queens generally mate with approximately 12–20 drones in managed and feral environments [21,28,29]. Queens mating only with a single or a small number of drones produce colonies that are less reproductively fit than those headed by queens mating with a larger number of drones [30,31]. Additionally, queens inseminated with sub-fertile drones (e.g., as a result of exposure to fipronil) are themselves reproductively impaired [32]. Thus, while “poor queens” may be a leading reason for colony failure, a major cause of queens being perceived as poor is very likely to be “poor drones” [32,33].

### 1.3. The Importance of Drones

Honey bee drones have a single purpose—to find a queen and mate with her [34]. This process involves being able to reach a drone congregation area (DCA), stay there long enough for a queen to arrive, locate a flying queen, out-compete thousands of other drones for copulation, and deliver sperm that the queen will store in her spermatheca [16]. As such, both the ability to copulate (largely a function of body size [35]) and the ability to inseminate (largely a function of sperm quality) are critical in order for a drone’s traits to be passed to the next generation. Drone semen can be negatively impacted by disease; [36,37] but see [38] and exposure to any number of agrichemicals [39,40,41], including commercial miticides intended to protect colonies from major disease vectors [42,43,44]. Factors that can be affected include spermatozoa concentration in the ejaculate or molecular qualities of the semen (e.g., ATP concentration), which reduce capacity and spermatozoa motility, and have been implicated in lower spermatozoa migration to the spermatheca or survival of stored spermatozoa [32]. Additionally, drones of varying sizes have been shown to differentially produce semen, with smaller drones producing less semen overall (but more spermatozoa/mg) while having less access to queens at DCAs [35,45,46] and generally being less representative at DCAs [47]. However, studies report a high degree of variability in spermatozoa counts among different drones [48] and different genetic lines of drones [49,50].

Colonies vary as to the fitness of the drones they produce; some colonies produce drones that are more likely to mate with a queen, and thus their genes are more represented in offspring [51]. It remains unclear, though, what factors maintain this variation among drones. Larval environment is critical for future drone reproductive health. Colonies that were protein-restricted reared drones that had a lower body mass, lower thorax mass, and smaller ejaculate volumes than those from colonies provided pollen [52]. Supplementing colony nutrition with sugar syrup and protein during spring growth periods resulted in colonies rearing heavier drones with larger thoraxes that produced a larger ejaculate volume, with higher spermatozoa viability [53]. Drone spermatozoa counts and everted semen volumes have been found to be higher earlier in the breeding season [49,50]. Colonies additionally adjust their drone production by season; this varies regionally, but the overall trend is that colonies primarily produce drones in the spring (when most queens are produced), peaking in early summer and declining as the season progresses [54]. However, larger colonies tend to produce more drones [55,56,57] and supplemental feeding can increase drone production [58], suggesting that environmental resource availability and not time of year is the primary factor determining drone rearing.

Drones also undergo significant senescence, initiating flight at around 10 days and living to an average of 30 days, regardless of when flight was initiated [59]. Additionally, several studies have suggested variation in drone sperm quality [49,60,61,62] at different ages and instrumental insemination success [63]. However, some colonies may produce drones that are more resistant to this senescence than others [49,50,64]. Older drones appear to tolerate a higher degree of oxidative damage [65], suggesting that spermatozoa count and body size are not the sole determinants of honey bee drone fitness.

### 1.4. Study Objective

Previous studies into how drones vary in their reproductive quality were often hampered by low sample size (as few as 5 individuals) [48], few time points determined arbitrarily or delineated broadly [49,50,62,66,67,68], or a lack of resolution (measuring spermatozoa viability or count exclusively) [60,64,67,68]. Without understanding how widely drones vary within a single colony and time, it is difficult to ascertain how impactful differences in drones due to population, season, or even disease and pesticide challenge really are to the mating environment. Honey bees exhibit complex behavioral and physiological changes as they age, leading to an emergent eusocial phenotype (reviewed in [69]). They additionally display extreme variations in lifespan based on reproduction division of labor [70]. However, comprehensive studies of the ontogeny of male honey bees are lacking [59]. We observed a large number of closely-related (brother) drones over the entire life span of the cohort. This study is therefore a critical first step to clarify our understanding of the variation of drone reproductive quality and ontogeny.

## 2. Materials and Methods

### 2.1. Drone Rearing and Collection

To control for any potential genotypic effects, standard-deep frames (243 mm × 480 mm) of comb containing drone pupae were removed from a single un-manipulated colony of European honey bees headed by a queen approximately 1 year old. Pupae were assessed for age by eye color and returned to the colony. 24 h before the estimated emergence time, the comb was removed to an incubator at 34 °C and 50% RH and placed with 100 worker bees collected from the brood nest of the source colony. Twice daily (8:00–9:00 and 16:30–18:00), emerged drones were collected into rearing boxes constructed of a wooden frame (127 mm × 127 mm × 25.4 mm), with queen excluder for sidewalls. To prevent any confounding effects of nest environment, each rearing cage was placed into one of 5 unrelated foster colonies for the duration of the experiment. Foster colonies were large colonies, headed by a naturally-mated queen, and utilized for no other purpose. A 4 cm insert was placed on the top-box and under the lid creating space to place the cages’ flat atop the frames. This enabled unfettered access to the drones without disrupting the hive structure. Each foster colony supported 4–7 cages of up to 124 drones apiece. Collection of newly emerged drones proceeded from 8 May 2017 to 17 May 2017, resulting in several cohorts of emerged drones spread amongst multiple rearing cages and foster colonies. Drones were then collected from rearing cages daily from 10 May 2017 to 16 June 2017 to achieve a sample from each chronological age from 0–30 days with a minimum of 4 h between each age and a final collection at 35 days. Cages pulled for each age was randomized, but there was an inescapable relationship among cage, age, and bank colony. However, because previous research has suggested that under normal circumstances (e.g., no injury to the drones), nest environment has little effect on adult drone physiology [64], we were justified in combining individuals from multiple foster colonies and cages for subsequent analyses. Drones were not permitted out of the cages and did not fly freely for the duration of the experiment; this was judged necessary to prevent sampling bias due to death due to flight or successful mating and has been shown previously to not affect drone development [67].

### 2.2. Dissection

Drones were collected from their rearing cages between 8:00–9:00 AM and brought into the lab, where they were immediately dissected. Because dissections took ~6 min to complete, some drones were out of the hive for up to 5 h prior to sacrifice. Individual drones were removed from their cages with soft forceps and placed on a plasticized dissection mat. Using a #12 sickle blade knife, the intersegmental membranes of the terga of the abdomen were pierced to prevent ejaculation. Drones were then placed in a chamber with CO_2_ sublimated from dry ice for 5–20 s. Unconscious drones were then weighed for whole body mass (M) to the nearest 0.1 mg. Drones were then placed onto the dissection stage (dorsal side up) and pinned using galvanized insect pins, at which point their heads and thoraxes were photographed. All four wings were clipped at the tegula with dissection scissors and pressed between two clear microscope slides, then photographed. Using curved dissection forceps, the 6th abdominal tergum was gripped, and scissors inserted into the sides of the abdomen, clipping the tergal segments free from the body on both sides. Gripping the sternal segments with curved forceps, the posterior abdominal cavity was partially pulled free to reveal the penis [16,71], mucus glands, and seminal vesicles. The mucus glands and seminal vesicles were carefully pulled free and placed on the dissection stage, then they were cut free from the ejaculatory duct with scissors. The mucus glands and seminal vesicles were then photographed and the seminal vesicles were cut free of the mucus glands for subsequent processing. The head and remaining abdomen were then cut away from the thorax and placed into separate 1.5 mL sterile, enzyme-free microcentrifuge tubes for subsequent processing. The thorax and legs were weighed (m) to the nearest 0.1 mg and similarly stored.

### 2.3. Semen Analyses

Freshly dissected seminal vesicles were placed into 1000 μL of buffer comprised of 3.0 g/L glucose, 4.1 g/L potassium chloride, 2.1 g/L sodium bicarbonate, and 24.3 g/L sodium citrate dihydrate. Vesicles were ruptured with forceps to release spermatozoa and the solution was mixed with the forceps and transferred via glass Pasteur pipet into an amber chromatography vial containing 10 μL of Syber 14 (Invitrogen Live/Dead spermatozoa staining kit #L7011; 1 mM in DMSO) diluted 1:500 into Dimethylsulfoxide (99.8%) and 10 μL propidium iodide solution (2.4 mM in water). The vial was screwed shut and gently vortexed 2 s @2000 rpm to homogenize and mix. Vials were allowed to sit a minimum of 5 min and a maximum of 4 h at room temperature to ensure uptake of the dyes into the cells. Samples were read using a Nexcelom Cellometer^®^ Vision Sperm Counter machine. 20 μL of the dyed spermatozoa were added to the Cellometer slide wells and each sample was counted for live and dead spermatozoa cells using the Cellometer Vision^®^ Software version 2.1.2.1 ©2018 Nexcelom Bioscience LLC (Lawrence, MA, USA). Exposure settings for the fluorescence was 1200 ms for the Syber and 7000 ms for the propidium iodide. Each sample was counted 3× in different locations and the resultant spermatozoa counts and viability percentages were averaged to provide a single estimate for each drone. Because only viable spermatozoa migrate from the vaginal chamber to the spermatheca [72], we used the number of viable spermatozoa as the viable spermatozoa count (C) in our analyses.

### 2.4. Digital Measurements

**Figure 1:**
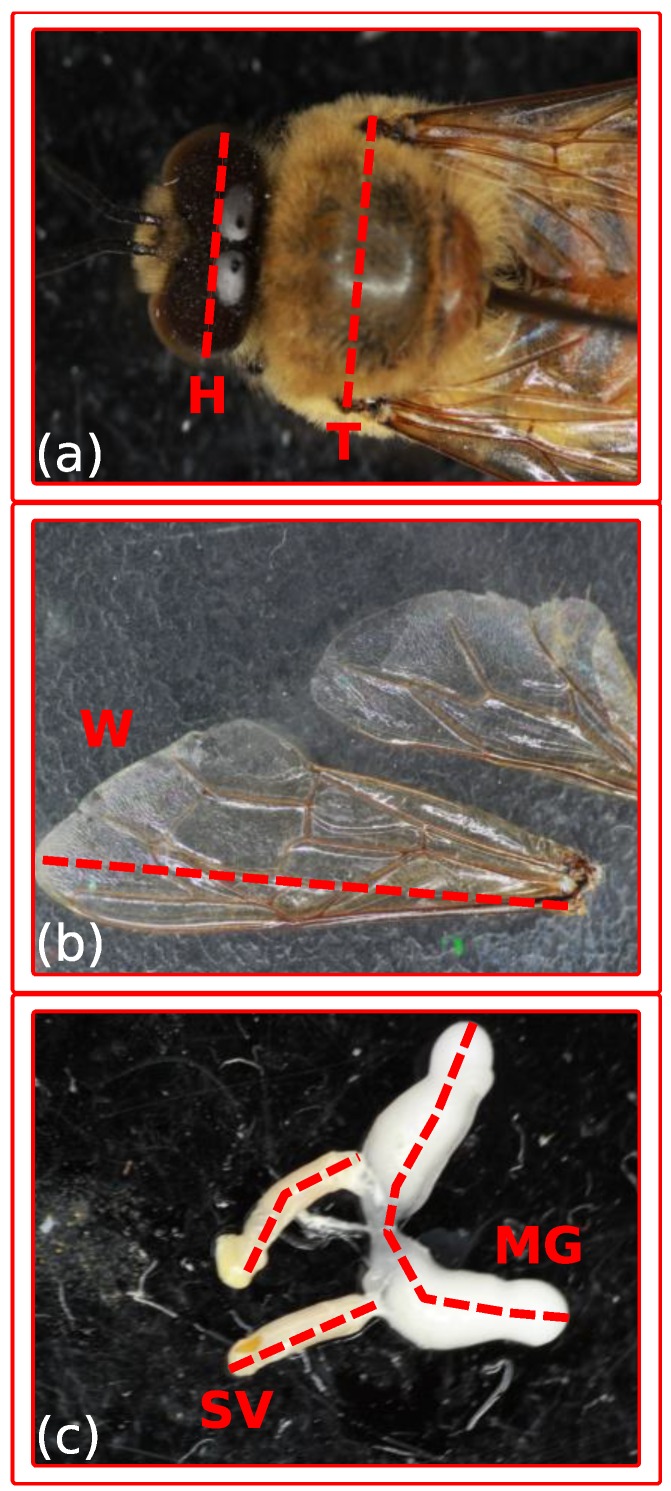
(**a**) measurement of drone heads (H) was perpendicular to the body axis at the widest point of the eyes and measurement of thorax width was measured as the distance between the tips of the two tegulae (T); (**b**) length of the dissected forewings (W) was taken as the length from the base of the costa to the tip of the wing, crossing through the intersection of the 2nd and 3rd sub-marginal cells with the 2nd marginal cell. The average of both wings was analyzed; (**c**) length of each mucus gland (MG) along the central axis was measured from the distal bulb to the ejaculatory duct. Lengths were averaged for analysis. Length of each seminal vesicle (SV) was measured along the central axis from the base of the vas deferens to the mucus gland and an average of both was used for analysis.

Images were processed using ImageJ version 1.51m9 [70]. Each image was calibrated to an image of a 1.00 mm glass ruler taken simultaneously under identical magnification and camera settings. The following measurements were recorded: the distance between the eyes perpendicular to the body axis at the widest point (Figure 1a, H); the width of the thorax as measured by the distance between the tips of the tegulae (Figure 1a, T); length of each forewing from the base of the costa to the wing edge, with the line crossing the intersection of the 2nd and 3rd sub-marginal cells with the 2nd marginal cell (Figure 1b, W); length of each seminal vesicle (Figure 1c, SV); and length of each mucus gland (Figure 1c, MG). Terminology was taken from Honeybee.drawwing.org [73].

### 2.5. Statistical Analyses

Statistical protocols are outlined in the results section. All analyses were performed in JMP Pro^®^ version 13.0.0 ©2016, SAS Institute, Inc. (Cary, NC, USA) Linear regression analyses followed the formula: $y=B_{0}+B_{1}x$, quadratic regressions followed the formula: $y=B_{0}+B_{1}x+B_{2}{\left(x-15.3152\right)}^{2}$.

## 3. Results

### 3.1. Migration of Spermatozoa to Seminal Vesicles

Presence of spermatozoa in the seminal vesicles was analyzed by logistic regression. Age was a significant factor explaining presence of spermatozoa following the equation $\ln\;\left(\frac{p}{1-p}\right)=B_{0}+B_{1}x$, where *B*_0_ = −2.85 ± 0.44, and *B*_1_ = 0.91 ± 0.12 (*Chi*^2^_1_ = 343.5; *p* < 0.0001; *r*^2^ = 0.70). The theoretical age at which 50% of drones initiate spermatozoa transfer to the seminal vesicles was 3 days, while 99% of drones should theoretically have initiated transfer by 8 days. In our direct observations, no individuals were found to have spermatozoa in the first day of life, whilst no drones were entirely absent of spermatozoa after 6 days.

### 3.2. Viable Spermatozoa Count

The effect of drone age on spermatozoa viability was tested using arcsin-transformed ratio of live spermatozoa: total spermatozoa. A linear model indicated no effect of age (*F*_1589_ = 0.267, *p* = 0.61, *r*^2^ = 0.00), however a quadratic model showed a significant relationship with spermatozoa viability increasing slightly to a theoretical maximum around 18 days followed by a slight decline (*F*_2588_ = 14.21, *p* < 0.0001, *r*^2^ = 0.046) following the quadratic formula where *B*_0_ = 1.02538 ± 0.01930, *B*_1_ = 0.0034 ± 0.0011, and *B*_2_ = −0.0006 ± 0.0001. Drones had a mean spermatozoa viability of 63.87 ± 1.13% (*N* = 671), which appears low until those drones without spermatozoa are excluded from the analysis, in which case the mean was 73.52 ± 0.80% (*N* = 522).

**Figure 2:**
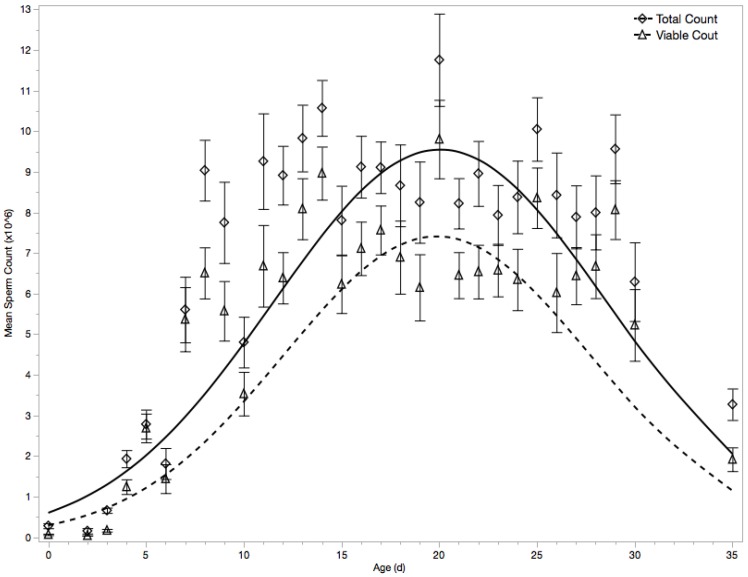
Newly emerged drones have no spermatozoa in their seminal vesicles. Counts of both total (diamond) and viable (triangle) spermatozoa significantly differ among differently aged drones (*p* < 0.0001). Total count was naturally higher than viable count, but there was no interaction effect between count type (*p* > 0.05). Peak counts occurred around day 19.

A similar result occurred when analyzing total spermatozoa count, with age effects on the natural-log-transformed values for spermatozoa count showed a rise to a maximum at around 20 days followed by a decline (Figure 2 *F*_2588_ = 128.64, *p* < 0.0001, *r*^2^ = 0.30) with a mean of 6.31 ± 0.18 million spermatozoa (including those without spermatozoa, *N* = 671) or 7.39 ± 0.19 million spermatozoa (excluding those without spermatozoa, *N* = 522) following the quadratic formula where *B*_0_ = 1.0573 ± 0.07840, *B*_1_ = 0.06380 ± 0.0046, and *B*_2_ = −0.0066 ± 0.0005.

Spermatozoa viability was highly correlated to spermatozoa count (*r* = 0.564; *p* < 0.0001). It was therefore of interest to ask whether spermatozoa viability changed because of age, or because of its correlation with spermatozoa count. This was tested by performing a multiple regression comparing the age effects on the total count of spermatozoa vs. the count of viable spermatozoa (i.e., those spermatozoa that were dyed with Syber 14, rather than propidium iodide). Again, the whole model was significant (Figure 2, *F*_71,174_ = 82.68, *p* < 0.0001, *r*^2^ = 0.33) as well as each age term (*Age*, *Age*^2^, and Age*Age^2^; *p* < 0.0001). Predictably, total count was significantly higher than viable count across all ages (Figure 2, *p* = 0.039). However, no interaction term including any age term and count was significant (Figure 2, minimum *p* = 0.461), supporting the decision to utilize viable spermatozoa count as a consolidated measure of drone spermatozoa health. Drones in this study contained a mean of 4.93 ± 0.15 million spermatozoa (including those without spermatozoa, *N* = 671) or 5.82 ± 0.16 million spermatozoa (excluding those without spermatozoa, *N* = 522) in their seminal vesicles.

### 3.3. Whole Body Mass & Thorax Mass

**Figure 3:**
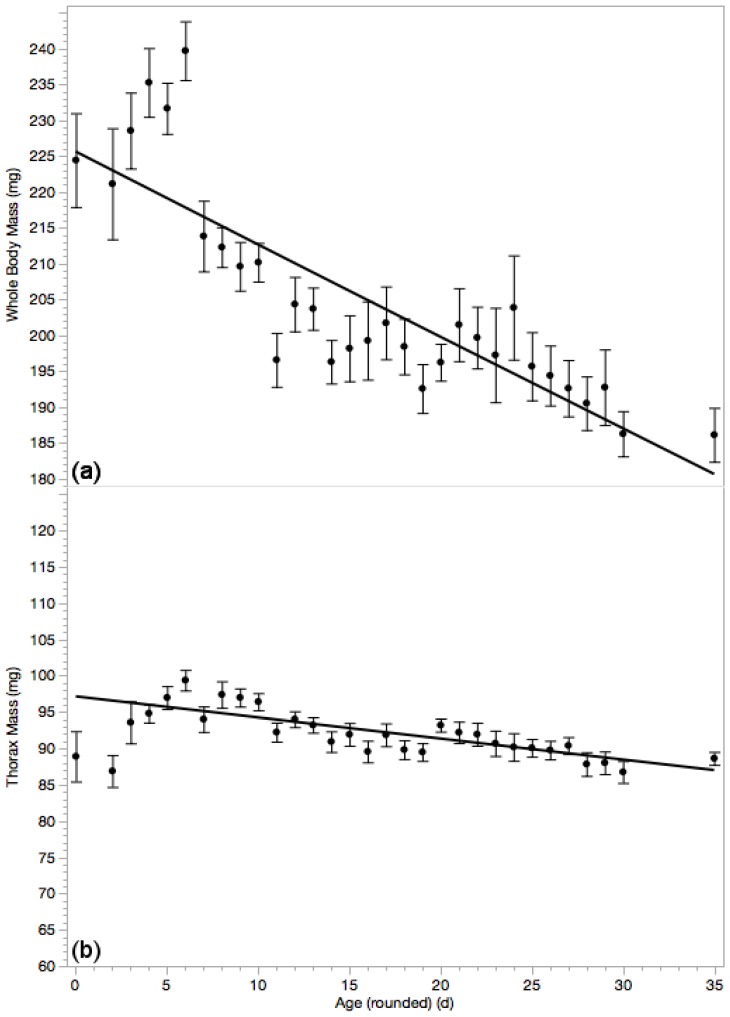
Whole body wet weights (**a**) was significantly smaller in older drones (*p* < 0.0001). There appears to be an initial increase in mass and a sudden drop after 6 days, perhaps the time when drones begin physiological preparation for flight. Thoracic mass (**b**) is also smaller in older drones (*p* < 0.05), though this trend is steadier.

Both whole body mass (Figure 3a, *F*_1669_ = 346.66; *p* < 0.0001; *r*^2^ = 0.341; *N* = 671; linear formula: *B*_0_ = 228.76 ± 1.42 and *B*_1_ = −1.47 ± 0.08) and thoracic mass (Figure 3b, *F*_1669_ = 91.09; *p* < 0.0001; *r*^2^ = 0.12; *N* = 671; linear formula: *B*_0_ = 96.15 ± 0.48 and *B*_1_ = −0.26 ± 0.03) were significantly smaller in older drones. Drones weighed an average of 206.28 ± 0.92 mg, with a mean thoracic mass of 92.22 ± 0.27 mg.

### 3.4. Body Measures

**Figure 4:**
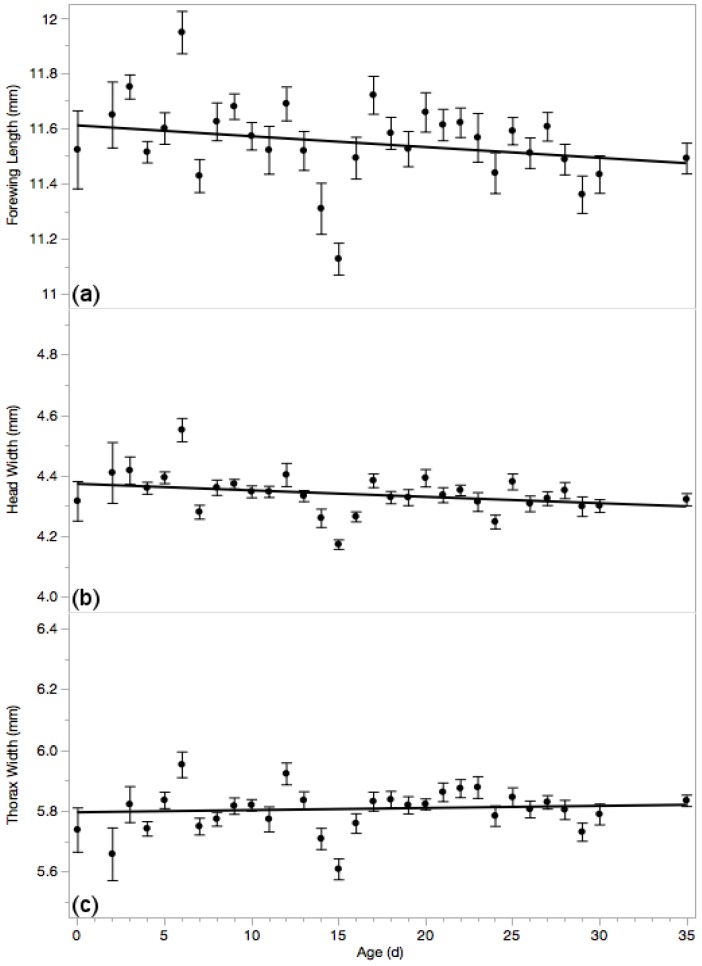
Forewing length (**a**) was significantly shorter in older drones (*p* < 0.001) as was head width (**b**) (*p* < 0.005). Thorax width (**c**) did not vary among differently aged drones (*p* > 0.05). Means ± standard errors are reported.

Forewing length was significantly shorter in older drones (Figure 4a, *F*_1667_ = 33.40; *p* < 0.0001; *r*^2^ = 0.05; *N* = 669; linear formula: *B*_0_ = 11.68 ± 0.03 and *B*_1_ = −0.008 ± 0.001) with a mean forewing length of 11.55 ± 0.014 mm.

Additionally, older drones had slightly, but significantly, narrower head widths than their younger brothers (Figure 4b, *F*_1659_ = 28.06; *p* < 0.0001; *r*^2^ = 0.041; *N* = 661; linear formula: *B_0_* = 4.38 ± 0.01 and *B*_1_ = −0.003 ± 0.00005). Drone heads averaged 4.343 ± 0.005 mm.

Distance between the tegulae did not significantly differ among differently aged drones (Figure 4c, *p* = 0.95) with an average width of 5.810 ± 0.005 mm (*N* = 661).

### 3.5. Reproductive Glands

**Figure 5:**
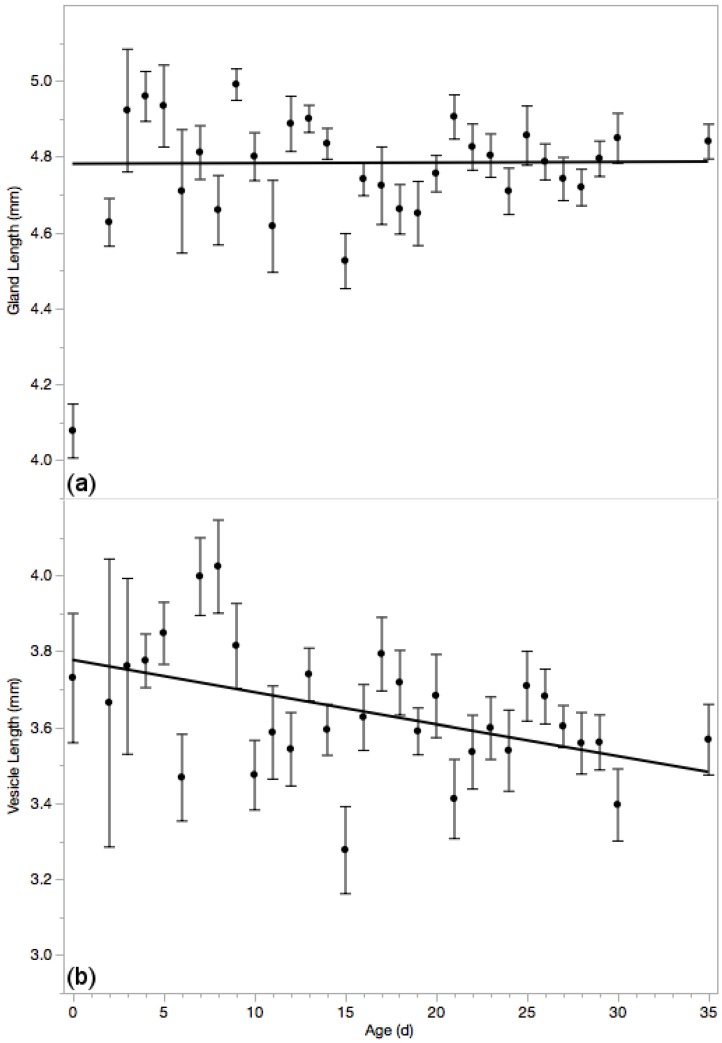
Mucus glands (**a**) visually increased in size over the first several days of life but did not significantly vary thereafter (*p* > 0.05) and was not smaller in older drones. Seminal vesicles (**b**) were significantly smaller in older drone (*p* < 0.05). Means ± standard errors are reported.

Mucus glands are visibly smaller and transparent in the first 3 days of life; after which they fill with accessory fluids over the course of a day or two. Otherwise, however, mucus glands are not significantly different among differently aged drones (Figure 5a, *p* = 0.11) with an average length of 4.716 ± 0.015 mm (*N* = 665).

Older drones had significantly shorter seminal vesicles (Figure 5b, *F*_1653_ = 18.66; *p* < 0.0001; *r*^2^ = 0.028; *N* = 655; linear formula: *B*_0_ = 3.73 ± 0.03 and *B*_1_ = −0.008 ± 0.002) with an overall average of 3.610 ± 0.017 mm. This result is predictable considering the vesicles are essentially tubes laden with spermatozoa and the high degree of correlation between spermatozoa count and vesicle length (*r* = 0.36; *p* < 0.0001).

### 3.6. Multivariate Analyses

Initial correlation of measured traits indicated a negative correlation of body mass and spermatozoa count (*r* = −0.14; *p* < 0.001; *N* = 591). This surprising result makes sense when the ontogeny of body mass in pre-flight drones is considered. The decrease in body mass, presumably due to flight preparation similarly to workers likely was the main cause of this. We therefore trimmed the dataset to eliminate all drones that were younger than 11 days as pre-flight drones. This time is based on the biodemographic data published by Rueppell et al. (2005) [59]. After excluding pre-flight drones, all measures were significantly and positively correlated with the natural log-transformed viable spermatozoa count, excepting head width; additionally, all body measures were significantly positively correlated with each other (Table 1).

**Figure 6:**
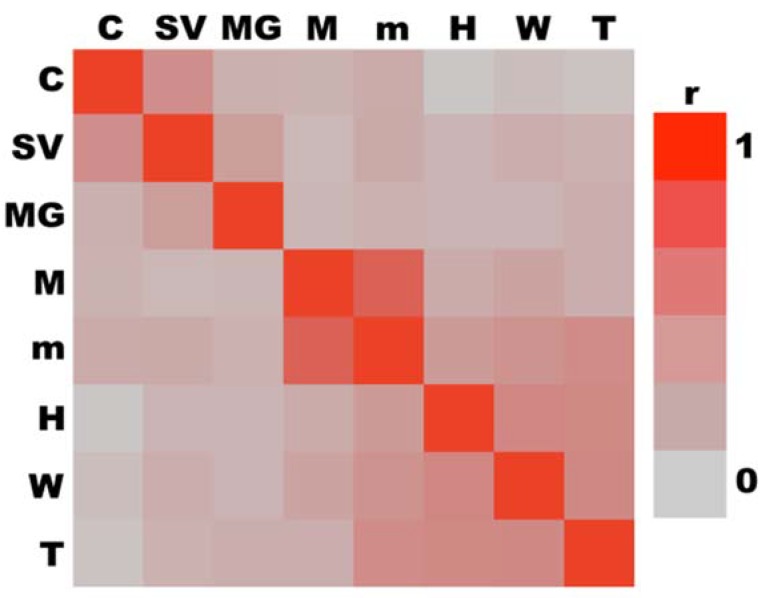
Drones aged 11+ d were analyzed for correlation. Viable spermatozoa count (C), seminal vesicle length (SV), mucus gland length (MG), whole body mass (M), thoracic mass (m), head width (H), forewing length (W), thoracic width (T). All measures were significantly positively correlated (*p* < 0.05) excepting C:H and C:T (*p* > 0.05). Two clusters were observed: (1) reproductive development (C, SV, & MG) and (2) body size (M, m, H, W, & T).

Correlations were clustered such that all variables within a cluster were more related to the resultant first principal component than those generated when included in alternate clusters by *r*^2^-comparison (Figure 6) [74]. This resulted in two clusters: (1) reproductive characteristics consisting of viable spermatozoa count, vesicle length, and gland length; and (2) body size measures consisting of body mass, thoracic mass, head width, forewing length, and thoracic width. The first principal component of each cluster was defined as “reproductive development” (Table 2) and “body size” (Table 3), respectively.

**Figure 7:**
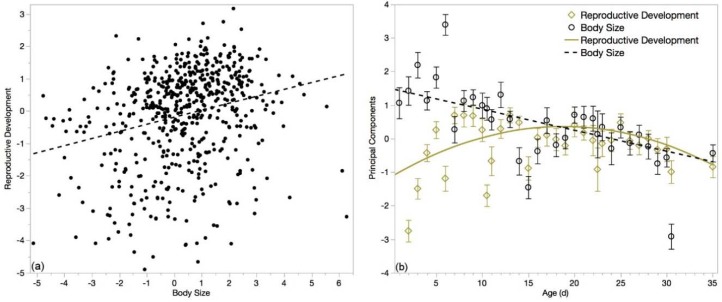
(**a**) Reproductive development, composed of the variables C, SV & MG, was significantly correlated with body size, composed of the variables M, m, H, T & W (*p* < 0.0001). Though the coefficients for the principal components were generated from flight-aged drones (11 + d), this correlation holds over the entire lifespan of drones; (**b**) Both principal components vary significantly by age (*p* < 0.05). Body size (black circle) varied in a relatively linear fashion, with older drones being smaller than younger drones. Reproductive development (yellow diamond) varied along a quadratic function, with drones aged 16 days being the most reproductively developed.

Within the selected age-range, body size and reproductive development principal components were significantly positively correlated (*r* = 0.36; *p* < 0.0001; *N* = 366), as expected given the correlation of the loading variables. Even when applying the principal component coefficients to the entirety of the age range, the components remain significantly, positively correlated (Figure 7a; *r* = 0.26; *p* < 0.0001; *N* = 574).

Principal components varied over age, both within the trimmed dataset and across the entire age range. Reproductive development decreased slightly in a linear manner over the flight-age range (*F*_1364_ = 4.19; *p* = 0.04; *r*^2^ = 0.011; *N* = 366; linear formula: *B*_0_ = 0.5695 ± 0.2270 and *B*_1_ = −0.0211 ± 0.0102). Over the entire age-range, reproductive development followed a quadratic function—which is expected, given the distribution of viable spermatozoa count over the same age—with a maximum at 16.8 days (Figure 7b; *F*_2585_ = 11.67; *p* < 0.0001; *r*^2^ = 0.04; *N* = 588; quadratic formula: *B*_0_ = −0.1868 ± 0.1356, *B*_1_ = 0.0214 ± 0.0080, and *B*_2_ = −0.0039 ± 0.0008). Body size decreased linearly over both the flight-age range (*F*_1364_ = 4.65; *p* < 0.05; *r*^2^ = −0.012; *N* = 366; linear formula: *B*_0_ = 0.6608 ± 0.2881 and *B*_1_ = −0.0280 ± 0.01298) and the full range (Figure 7b; *F*_1659_ = 107.35; *p* < 0.0001; *r*^2^ = 0.14; *N* = 661; linear formula: *B*_0_ = 1.634 ± 0.1237 and *B*_1_ = −0.0712 ± 0.0069). Behavior of the principal components followed similar trends as the loading variables.

## 4. Discussion

### 4.1. Spermatozoa Migration

In our observations, no drones had semen present in their seminal vesicles on the first day of life. This percentage increased to about 50% of the drones by day 3, with spermatozoa transfer to the seminal vesicles begun in all drones by day 6. Drones are widely and often described as completing their semen production and spermatozoa maturation during the first week of adult life [61,75,76,77,78,79,80]. However, previous empirical evidence for this assertion appears to be limited. To our knowledge, ours is the first study to directly demonstrate the timing of the migration of spermatozoa to the seminal vesicles using highly controlled age groups [67,68].

### 4.2. Drone Senescence

Honey bee drones undergo senescence as adults, with lifespans being apparently limited to about 30 days regardless of whether they fly [59] or not (our current observations). However, we did not see the decrease in spermatozoa viability in older drones reported by Stürup et al., 2013 [64] when we accounted for variations in spermatozoa count. This could be a result of the increased sample size in our study (671 drones over 32 time points in our study vs. 144 drones over 7 time points and 5 colonies in theirs) or perhaps their explanation that some colonies do not show this mode of senescence means that our single colony was particularly resistant to spermatozoa death. It is also possible that dead spermatozoa are being degraded and reabsorbed and therefore we do not observe the decreased viability. In any event, our results suggest that a major mode of reproductive senescence (death of the spermatozoa) is more complicated than previously suggested and, all things being equal, semen from older drones is unlikely to be less viable than that from younger drones. Because we did not measure any other sperm parameters (e.g., motility or oxidative damage), it remains possible that semen or spermatozoa from older drones is in some other way compromised.

### 4.3. Small Drones

We observed that older drones were in many ways smaller than their younger brothers, even within the flight-aged range. Whole body wet weights were significantly smaller, which can be expected even in caged bees. In honey bee workers and queens, there is a significant loss in body weight as the switch to flight to initiate foraging or swarming (respectively) occurs [16]. Though drones in our study were not allowed to fly, it is reasonable to suggest that much of the weight difference between pre-flight and flight-aged drones is a result of such physiological changes. Further, the caged drones eventually defecate, either within the hive or in transit to the laboratory; also contributing to weight loss.

The differences in thoracic mass is harder to explain. Because the thorax is comprised almost entirely of muscle, the mass loss is likely to be due to decreased muscle development, which in turn could be a result of degradation from starvation or maltreatment. Though we observed workers attending drones even late in life, there were increasing signs of aggression in older drones (e.g., wing damage) perhaps suggesting that drones were no longer being fed enough to survive, or otherwise being ill-treated. This may also explain the sudden increase in death, though as we did not directly measure how many drones died over the course of the study, any conclusions of mortality must be cautious.

It is more difficult to speculate on body measures that we would not expect to be different (e.g., forewing length and head width were smaller in older drones). One possibility is a bias in collecting the largest drones early in life; because we caged several cohorts of drones and pulled from cages up from 1–4 times (median 2), this cannot entirely be discounted. However, because we pulled from 17 cages, we find it unlikely that this bias is significantly impacting our results. Another is variations in the time of emergence. Previous research suggests that drone morphological and reproductive measures change over the course of the season [49]. However, because all drones in this study emerged within the same 10-day period, we consider this explanation unlikely. Alternatively, our observations are consistent with the hypothesis that smaller drones simply live longer, and that when we sample aged drones, we are more likely to sample smaller—and therefore somewhat less fecund—drones. A negative relationship between reproductive maturation and lifespan is well-established in other taxa, with females in the social Hymenoptera being a rare exception [81]; it is possible that drones follow the more common trend.

Smaller drones are unlikely to be simply worker-laid; about 9% of drones at DCAs have been observed to be small [35], while worker-laid drones only make up approximately 0.12% of live drones [82]. How “small” is defined and natural variation of body size among populations makes comparisons difficult, but it is reasonable to suggest that small drones are produced by queens as part of normal colony reproductive function. The persistence of smaller drones in the colony in light of the observed penalty to mating success demands an explanation. Previous explorations into the fitness advantages of smaller drones have found equivocal evidence that small drones may fly at different times of day [35,83,84]. Successful mating can occur well outside peak mating seasons and times. It has been shown that seasonal variation in drone production is such that less-fertile drones are produced outside of mating peaks [49]. Lifespan differences would be one potential mechanism for smaller drones acting in these tails. Drones with different ontogenic or behavioral traits may represent a low-cost, high-risk reproductive strategy.

### 4.4. Effect on Queen Mating Success

Our findings have bearing on the reproductive potential and longevity of honey bee queens, although further studies will be required as to how drone quality affects queen quality. Given the extensive variation in drone size, sperm count, and other critical measures of fitness, it is unclear how this variation influences spermatozoa representation in a queen’s spermatheca. We speculate that there may be trade-offs in different tokens of drone fitness. For example, larger drones may have an advantage in copulation success, but smaller drones may have an advantage in lifetime longevity. Nonetheless, the dynamics by which these reproductive traits are beneficial at the drone, queen, and colony level warrant further investigation.

## 5. Conclusions

Our study shows that drones produced by a well-functioning colony vary to a larger degree than previously reported. It is therefore likely that sample sizes used in earlier studies obfuscated some of these associations. Further, drones vary significantly by age, though the major result of this variation manifests in very young or very old drones; thus “flight-aged” drones may be taken as equivalent for the purposes of morphometric and semen analyses. Older drones were significantly smaller and less reproductively robust than their younger, flight-aged siblings. We speculate that this is the result of a variation in survivorship (e.g., smaller drones could be developmentally delayed and therefore live longer). It will be necessary to pair non-destructive sampling along with individual-level observations to determine the interactions between body size, reproductive, and behavioral ontogeny among multiple genetic and environmental contexts.

## Figures and Tables

**Table 1:**
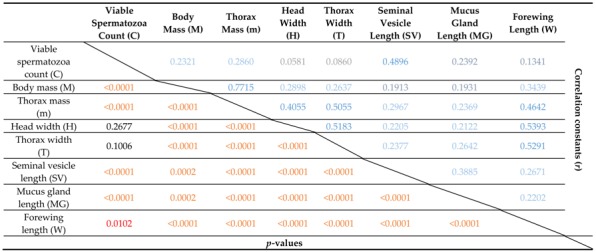
Correlation Matrix ^1^.

^1^ Correlation matrix of body and reproductive measures. The upper half of the matrix (blue shading) represents the correlation coefficients (*r*) while the lower half (orange shading) presents the *p*-values for those coefficients.

**Table 2:** Reproductive Development ^1^.

Loading Variable	Eigenvector
Viable spermatozoa count (C)	0.576
Seminal Vesicle length (SV)	0.638
Mucus Gland length (MG)	0.511

^1^ The first principal component related to reproductive development explained 58.5% of the variation in the loading variables (eigenvalue 1.75).

**Table 3:** Body Size ^1^.

Loading Variable	Eigenvector
Body mass (M)	0.418
Thorax mass (m)	0.498
Head width (H)	0.426
Thorax width (T)	0.441
Forewing length (W)	0.450

^1^ The first principal component related to body size explained 57.3% of the variation in the loading variables.
